# Clinical efficacy and safety of ultrasound-guided injection with low-molecular-weight peptides from hydrolyzed collagen in patients with first carpometacarpal joint osteoarthritis

**DOI:** 10.3389/fmed.2026.1891026

**Published:** 2026-07-20

**Authors:** Marin Petric, Marco Di Carlo, Luca Latini, Riccardo Mashadi Mirza, Francesco Porta, Florentin Ananu Vreju, Emilio Filippucci

**Affiliations:** 1 Department of Rheumatology and Clinical Immunology, School of Medicine Split, University Hospital Split, Split, Croatia; 2 Rheumatology Unit, Department of Clinical and Molecular Sciences, Polytechnic University of Marche, Ancona, Italy; 3 Centro Medico e Fisioterapia “Salute e Benessere”-Senigallia, Ancona, Italy; 4 Radiology Unit, Riccione-Cattolica Hospital, Rimini, Italy; 5 Interdisciplinary Pain Medicine Unit, Santa Maria Maddalena Hospital, Occhiobello, Italy; 6 Department of Rheumatology, University of Medicine and Pharmacy Craiova, Craiova, Romania

**Keywords:** collagen peptides, first carpometacarpal joint, hand osteoarthritis, hydrolyzed collagen, intra-articular therapy, ultrasound-guided injection

## Abstract

**Background:**

First carpometacarpal (CMC-1) joint osteoarthritis (OA) is a common and disabling condition associated with pain, reduced pinch strength, impaired hand function, and progressive limitations in activities of daily living. Although conservative treatment remains the first therapeutic step, the optimal injectable option for patients with persistent symptoms is still debated.

**Objective:**

To evaluate the clinical efficacy and safety of ultrasound (US)-guided intra-articular injection with low-molecular-weight peptides (LWPs) derived from hydrolyzed collagen in patients with symptomatic CMC-1 OA.

**Methods:**

This protocol describes a prospective single-arm interventional study enrolling patients with symptomatic and radiographically confirmed CMC-1 OA. All eligible participants will undergo US-guided intra-articular injection with LWPs. The primary endpoint will be the change in pain during activity after 3 months. Secondary outcomes will include pain at rest, hand disability, grip and key pinch strength, rescue analgesic consumption, patient-reported improvement, ultrasonographic findings, treatment-related adverse events, and pain during activity and at rest after 1, 3 and 6 months.

**Expected results:**

The study is designed to assess whether the investigational treatment is associated with reduction in pain, improvement in hand function, and an acceptable safety profile over a follow-up period of up to 6 months. A secondary exploratory aim is to identify baseline clinical and US patterns potentially associated with clinical and imaging response.

**Conclusions:**

If supported by the study findings, US-guided intra-articular administration of LWPs may represent a promising minimally invasive therapeutic option for patients with CMC-1 OA who remain symptomatic despite standard conservative care.

## Introduction

Osteoarthritis (OA) of the first carpometacarpal joint (CMC-1), also referred to as thumb-base OA, is among the most common degenerative disorders of the hand and a major source of pain, weakness, and disability in middle-aged and older adults (1–5). Population-based studies show that radiographic CMC-1 OA is common, increases with age, and is more prevalent in women, particularly after menopause (1–3). Occupational and biomechanical exposures involving repetitive pinch, grasp, or thumb loading have also been associated with disease occurrence or progression (4, 5). Clinically, patients typically report pain at the base of the thumb, weakness during pinch and grasp, morning stiffness, crepitus, and progressive limitation in activities such as opening and holding jars, turning keys, handling small objects, or playing a musical instrument (5).

The pathophysiology of CMC-1 OA is multifactorial and includes progressive cartilage degeneration, synovial inflammation, capsuloligamentous laxity, osteophytes formation, and altered joint biomechanics leading to instability and pain (5–7). Imaging studies suggest that thumb-base OA represents a distinct phenotype within hand OA; structural damage, especially osteophytes, may be more strongly associated with pain than inflammatory changes alone (6, 7). Moreover, inflammation plays an important role in this kind of OA when compared to other kinds of OA. In fact, increased synovial and/or capsular vascularization in CMC-1 OA, as a sign of the joint inflammation also contributes to pain severity (7–9). These structural and biological changes contribute to persistent pain, deterioration in hand performance, and reduced quality of life. Standard conservative care is generally stepwise and includes patient education, activity modification, orthoses, pharmacological pain control, and supervised rehabilitation, while intra-articular injection is often considered before surgery when conservative therapy is insufficient (5, 10–13).

Injectable treatments currently used in clinical practice include corticosteroids, hyaluronic acid (HA), and other biological or viscoelastic formulations, but their relative efficacy remains heterogeneous across studies (10–13). In a randomized single-masked study, Monfort et al. reported differential short- and mid-term effects of HA and corticosteroid injections in CMC-1 OA (10), while the RHIZ'ART program by Cormier et al. further explored whether combining HA with corticosteroids improves outcomes compared with corticosteroids alone (11, 12). A recent systematic review and meta-analysis also highlighted the persistent uncertainty surrounding the magnitude and durability of benefit from corticosteroid injections in CMC-1 OA (13). In parallel, interest in collagen-based injectable medical compounds has grown. Reviews of intra-articular collagen therapies and broader collagen derivatives in OA suggest potential symptomatic and structure-supporting effects, although the evidence base remains heterogeneous and still limited (14, 15). Preclinical and clinical studies in knee OA indicate that injectable hydrolyzed collagen may modulate the intra-articular environment, support extracellular matrix homeostasis, and improve pain and function (16–19). According to in-vitro studies, chondrocytes presented significantly smaller cell density and greater matrix deposition after treatment with hydrolyzed collagen (16). Furthermore, high deposition of type-II collagen and inhibition of type-I collagen deposition were observed (16). In animal models, treatment with hydrolyzed collagen decreased levels of pro-inflammatory cytokines, parameters of oxidative stress, as well as cartilage degradation markers (19).

Although most available evidence derives from knee osteoarthritis, similar biological mechanisms may be relevant in small joints, although joint-specific differences should be considered. Evidence in thumb-base OA remains scarce, but recent data on collagen-based filler injection reported encouraging long-term clinical outcomes (20). Hydrolyzed collagen-derived low-molecular-weight peptides (LWPs) have therefore attracted increasing interest as a potentially safe, biologically active option for local treatment (15, 16, 21, 22). In addition, ultrasound (US)-guided LWPs injection has shown preliminary clinical benefit in another degenerative musculoskeletal condition, namely partial supraspinatus tendon tears (23).

US guidance is particularly relevant for the CMC-1 joint because of its small size and complex anatomy. Real-time imaging may improve needle placement accuracy, reduce the risk of extra-articular injection, and enhance procedural standardization. In addition, US enables characterization of joint inflammatory and structural lesions using validated scoring frameworks relevant for hand OA (6, 7, 24, 25). On this basis, the present manuscript proposes a clinical study designed to investigate the efficacy and safety of US-guided intra-articular injection with LWPs in patients with symptomatic CMC-1 OA.

## Rationale and hypothesis

The scientific rationale for this study is grounded in the hypothesis that LWPs may induce a beneficial local effect within the OA joint by supporting matrix turnover, improving the biological milieu, and contributing to symptom control. Experimental and translational studies on collagen derivatives suggest effects on extracellular matrix remodeling, chondrocyte behavior, and inflammatory pathways, while early clinical studies support good tolerability and potential symptomatic benefit after local or intra-articular administration (15–23). Because CMC-1 OA frequently affects fine motor performance and daily functioning, even modest improvements in pain and pinch-related function may have clinically meaningful consequences.

The primary hypothesis is that US-guided intra-articular injection with LWPs will result in clinically meaningful improvements in both resting and activity-related thumb pain at 3 months, while maintaining good local and systemic tolerability.

As secondary outcomes, improvements at 1, 3, and 6 months after the injection are expected in terms of disability assessed with patient-reported measures, grip and key pinch strength, US findings, and a reduction in analgesic use, in the absence of significant adverse events in the weeks following the procedure.

In third instance, this study will seek to identify potential clinical and imaging biomarkers that could predict better outcomes with LWPs injections.

## Methods

### Study design

This research is conceived as a multicentric prospective single-arm interventional study. Participants with symptomatic and radiographically confirmed CMC-1 OA will receive US-guided intra-articular injection with LWPs. The follow-up period will extend to 6 months; further extensions will be additionally discussed.

### Study setting and population

The study will be conducted in specialized outpatient settings dedicated to musculoskeletal care. Adults with persistent symptoms despite standard conservative management will be screened for eligibility. Radiographic assessment will be used to confirm diagnosis and to stage the disease severity.

## Eligibility criteria

Eligibility will be established on the basis of clinical, radiographic, and procedural criteria. Table 1 summarizes the proposed inclusion and exclusion criteria.

**Table 1:** Proposed eligibility criteria for enrollment.

Inclusion criteria	Exclusion criteria
Age ≥40 years	Prior thumb base surgery on the affected side
Symptomatic CMC-1 joint OA for over 3 months (joint pain, joint stiffness, decreased mobility, deformity, instability, functional impairment)	Intra-articular injection in the target joint within the previous 3 months
Radiographic confirmation of CMC-1 joint OA using the Eaton-Littler classification	The Eaton-Littler classification grade IV
Pain at the thumb base for at least 3 months	Inflammatory arthritis or crystal arthropathies with treatment changes during the previous 3 months or planned treatment changes during follow-up
Insufficient response to standard conservative treatment	Active local or systemic infection
Average pain intensity ≥4/10 during hand use in the week before enrollment as assessed by NRS for pain	Known hypersensitivity to collagen-derived products or study excipients
Ability to provide written informed consent	Coagulation disorder or anticoagulation judged_incompatible with the procedure
	Pregnancy or breastfeeding
	Neurological or musculoskeletal conditions that could interfere with outcome assessment: carpal tunnel syndrome, ulnar neuropathy, cervical radiculopathy, peripheral neuropathy, multiple sclerosis, stroke, motor neuron disease and Parkinson disease, ligament injuries (e.g., Skier's Thumb) and Dupuytren disease
	History of severe hand and thumb trauma
	Impossibility of safe US-guided injection as assessed by the investigator
	US visible extravasation of the injectate LWPs outside the joint space during the procedure

CMC-1, first carpometacarpal joint; OA, osteoarthritis; NRS, numerical rating scale; US, ultrasound.

## Intervention

Since this is a multicenter study, to maximize reproducibility, the protocol will provide a detailed description of patient positioning, skin antisepsis, US transducer orientation, needle caliber, approach to the joint, injected volume, and post-procedural recommendations. Moreover, an US calibration exercise will be performed by the sonographers of each center involved in the study. The exercise will be based on a shared database of representative US images. Each sonographer will be asked to score US images in terms of inflammatory and structural findings using semiquantitative scales (0–3) according to the OMERACT hand OA US definitions and semiquantitative scoring system, thereby improving inter-reader standardization before recruitment (24, 25).

Before enrollment, participants will complete visual analog scale (VAS) for pain at rest and during activity, and the Duruöz Hand Index (DHI) and Quick Disabilities of the Arm, Shoulder and Hand (QuickDASH). Grip strength and key pinch strength will be measured with calibrated dynamometer and pinch gauge. To improve comparability across visits and centers, the same device model should be used within each center throughout follow-up and the device characteristics should be recorded a priori.

Baseline US assessment will include the detection and semiquantitative scoring of synovial hypertrophy/effusion, power Doppler signal, and osteophytes, assessed with a high-resolution probe (B-mode frequency >15 MHz; pulse repetition frequency <1.0 kHz) using previously described and validated scanning protocols for hand OA (25).

## US-guided injection procedure

All procedures will be performed by an experienced physician trained in musculoskeletal US-guided interventions. The injection will be performed with hand placed on a solid surface with the palm facing upward. A high-frequency linear transducer will be used to identify the CMC-1 joint and to guide needle placement in real time in-plane approach to ensure more precise injection. A single US-guided intra-articular injection of 1 mL of LWPs will be administered into the CMC-1 joint through a 23-gauge needle. Correct intra-articular delivery will be confirmed using US guidance. This approach is methodologically justified in this joint because thumb CMC injections are technically demanding and have lower landmark-based accuracy than several other common hand injections (26).

## Outcome measures

The primary efficacy endpoint will be the change in pain intensity during activity, measured on a 0–10 NRS (numerical rating scale), from baseline to 3 months. A reduction of 2 points on a 10-point numerical scale is considered a clinically significant improvement in the musculoskeletal setting. Secondary endpoints will include pain at rest, DHI and QuickDASH scores, grip and key pinch strength, rescue medication use, patient global assessment, ultrasonographic findings, and adverse events. This choice is consistent with hand OA trial recommendations, which emphasize pain, physical function, patient global assessment, and—for longer studies—imaging outcomes (27, 28). Table 2 provides a summary of the assessment schedule.

**Table 2:** Proposed schedule of interventions and follow-up assessments.

Assessment	Baseline	1 Month	3 Months	6 Months
Eligibility and consent	X			
NRS for pain at rest and during activity	X	X	X	X
DHI and QuickDASH	X	X	X	X
Grip and key pinch strength	X	X	X	X
US assessment	X		X	X
Injection procedure	X			
Rescue medication review		X	X	X
Adverse events		X	X	X
Patient global assessment/satisfaction		X	X	X

NRS, numerical rating scale; DHI, Duruoz Hand Index; QuickDASH, shortened Disability of Arm, Shoulder and Hand Questionnaire; US, ultrasound.

## Concomitant treatment

Stable non-pharmacological treatment, including orthoses and prescribed hand exercises, may be continued provided that these measures are documented and remain substantially unchanged during the main assessment phase. Rescue analgesia with acetaminophen/paracetamol and/or non-steroidal anti-inflammatory drugs may be allowed and recorded in patient diaries. Moreover, to minimize confounding effects, concomitant treatments will be kept stable throughout the study period whenever possible and any changes will be carefully recorded and considered in the analysis. The use of additional injections or surgical procedures targeting the study joint during follow-up should be treated as treatment failure or major protocol deviation.

## Sample size

Because the present protocol describes a single-arm prospective study, the sample size should be based on feasibility and precision of estimation rather than on a between-group superiority hypothesis. The current between-group calculation is therefore not methodologically aligned with the revised design. The final sample size should be determined with dedicated biostatistical support once the primary outcome metric, its expected variability, and the target precision or clinically important change have been definitively specified (25).

## Statistical analysis

Analyses will be performed according to a predefined statistical analysis plan. Because the protocol is currently single-arm and non-randomized, the primary analytical population should include all enrolled participants who receive the injection and provide at least one post-baseline assessment. A per-protocol analysis may be performed as a sensitivity analysis.

Descriptive statistics will summarize baseline demographic, clinical, radiographic, and ultrasonographic characteristics. The primary endpoint—change in pain during activity from baseline to 3 months—may be analyzed with a paired-samples approach or, preferably, with a mixed-effects repeated-measures model including time as a fixed effect and participant as a random effect. Continuous secondary outcomes may be examined with similar longitudinal models, whereas categorical responder analyses may be reported descriptively or explored with appropriate regression methods if sample size permits. Missing data should be handled according to a prespecified strategy consistent with the amount and mechanism of missingness. If sample size allows, exploratory analyses may be performed to assess whether baseline clinical, radiographic, or US features are associated with clinical response (27).

## Safety monitoring

Safety surveillance will include systematic recording of local and systemic adverse events throughout the study. Particular attention will be paid to post-injection pain flare, swelling, bruising, local inflammatory reaction, hypersensitivity, infection, neurovascular injury, and clinically meaningful worsening of symptoms. Serious adverse events will be reported according to local institutional and regulatory requirements.

## Ethics and data management

The study will be conducted in accordance with the Declaration of Helsinki, Good Clinical Practice principles, and applicable national regulations. Written informed consent will be obtained from all patients before any study-specific procedure. Ethics committee or institutional review board approval will be secured before the start of recruitment. Study data will be collected through standardized case report forms and stored in a secure database with controlled access.

## Expected results and clinical impact

The proposed intervention may provide clinically relevant pain improvement, better pinch and grip-related function, measurable changes in patient-reported hand disability and intra-articular power Doppler signal. These domains are particularly relevant in CMC-1 OA because disease burden often becomes evident in routine activities rather than in isolated pain scores alone. In addition, the study may help identify baseline clinical, US, and/or radiographic findings associated with better outcomes after LWPs injection.

If the intervention demonstrates both efficacy and safety, it could expand the range of minimally invasive options available to patients with persistent symptoms who are not yet candidates for surgery or who wish to postpone operative treatment. The results obtained in our cohort of patients injected with LWPs will be interpreted in the context of previously published studies, such as RHIZ'AR by Cormier et al. (9, 10).

## Discussion

Thumb base OA remains a frequent therapeutic challenge because symptoms may be substantial regardless of radiographic severity, and because patients often seek improvement in dexterity and pinch performance in addition to pain relief. In this context, a targeted injection strategy supported by biological plausibility and precise US guidance warrants formal clinical evaluation.

The present protocol has several strengths. First, it focuses on a clinically relevant and anatomically specific joint that is often underrepresented in larger OA trials. Second, the use of US guidance and a pre-study sonographer calibration procedure improve procedural consistency and internal validity. Third, the protocol incorporates both symptom-based and function-based outcomes together with objective strength measures and structured safety monitoring. Fourth, the use of disease-relevant assessment scales specifically designed for hand OA improves comparability with analogous studies and with current methodological recommendations (27, 28). Fifth, the multicentric and prospective nature of the study improve external validity and the generalizability of the findings.

Potential limitations include the absence of a control arm, the lack of blinding, and the exploratory sample size, all of which may limit causal inference and external validity. Moreover, a 6-month follow-up may be adequate for symptomatic assessment, but remains insufficient to evaluate structural disease modification. If the investigators intend to make formal comparative claims against other injective strategies, a randomized controlled design would be required. Accordingly, the present protocol should be interpreted as a prospective interventional study aimed primarily at generating structured efficacy and safety data and refining the design of future controlled trials. This design is methodologically more consistent with the current protocol rather than a parallel-group randomized one, which would require a prespecified comparator arm, allocation procedure, and blinding strategy (27).

## Conclusion

This manuscript outlines a protocol to investigate the clinical efficacy and safety of US-guided intra-articular injection with LWPs in patients with symptomatic CMC-1 OA. By integrating imaging-guided delivery, clinically meaningful endpoints, disease-specific functional measures, and structured safety monitoring, the study is designed to generate practical preliminary evidence on a minimally invasive treatment strategy for thumb base OA.

Results from this study may be interpreted alongside the published literature on intra-articular therapies for rhizarthrosis, including corticosteroids and hyaluronic acid, but any comparison with external cohorts will remain descriptive rather than inferential. The study may also help identify baseline clinical, ultrasound, and/or radiographic findings associated with better outcomes after LWPs injection, thereby informing the design of subsequent controlled studies.
